# Gender Differences in the Association between Physical Activity and Mortality in Chronic Kidney Disease: Results from the National Health and Nutrition Examination Survey (2011–2018)

**DOI:** 10.3390/jcm12030779

**Published:** 2023-01-18

**Authors:** Wei Peng, Min Han, Gang Xu

**Affiliations:** Department of Nephrology, Tongji Hospital, Tongji Medical College, Huazhong University of Science and Technology, Wuhan 430030, China

**Keywords:** chronic kidney disease, physical activity, mortality, gender difference

## Abstract

Background: Physical activity is indispensable in the management of chronic kidney disease (CKD). The aim of this study was to investigate gender difference in the association of physical activity with mortality among the CKD population. Methods: In total, 3701 participants with CKD from the 2011 to 2018 NHANES with linked mortality data were classified into different groups based on the intensity of self-reported physical activity. Multivariable-adjusted Cox proportional hazards models were used to examine the associations between physical activity and mortality. Results: During the median follow-up of 53.7 months, 694 all-cause deaths and 226 cardiovascular deaths were recorded. Patients were categorized into extremely highly active (>1500 MET-min/week), highly active (>600, ≤1500 MET-min/week), low-active (>0, ≤600 MET-min/week), or inactive (0 MET-min/week) groups. Among males, the multivariable Cox regression showed that the low-active group (HR, 0.67; 95% CI, 0.48–0.93) and highly active group (HR, 0.60; 95% CI, 0.41–0.88) were independently associated with lower risks for all-cause mortality, compared to the inactive group. The risks of all-cause mortality did not further decrease once physical activity surpassed 1500 MET-min/week, indicating a U-shaped association in males. In females, only the extremely highly active group (>1500 MET-min/week) was significantly associated with a mortality risk compared to inactivity (HR, 0.59; 95% CI, 0.39–0.89). Conclusions: Any amount of physical activity is associated with reduced all-cause mortality in male CKD participants, while in female patients, only the extremely highly active group shows the significant association.

## 1. Introduction

Chronic kidney disease (CKD) is an increasing global health burden due to high morbidity and mortality [1]. The role of physical activity on the prevention of mortality and clinical adverse events in the CKD population has been well established [2]. Specifically, in patients with advanced CKD, higher levels of physical activity could reduce all-cause mortality by 50% [3]. In patients with dialysis, greater exercise frequency was found to be associated with longer survival and higher health-related quality of life [4]. Thus, physical activity has been recognized as a key modifiable lifestyle factor in the management of patients with CKD. In 2012, Kidney Disease: Improving Global Outcomes (KDIGO) guidelines recommended patients with CKD undertake moderate physical activity for at least 30 min, five times per week [5]. Since then, physical activity has become an indispensable component in CKD management.

Sex-based differences are gradually known to exist in the CKD population. For example, despite a higher prevalence of CKD among women, women tend to experience a slower decline in renal function and more men progress to end-stage renal disease [6,7]. As for related pathophysiology, it is reported that oxidative stress and nitric oxide deficiency may contribute to the gender disparity in CKD [8,9]. To our knowledge, few reports were conducted to investigate gender disparities in the association between physical activity and survival in the CKD population. Therefore, we examined the 2011–2018 National Health and Nutrition Examination Survey (NHANES) database, and evaluated the dose–response association between physical activity and mortality; and possible gender differences in this relationship.

## 2. Materials & Methods

### 2.1. Data Source

**Figure 1:**
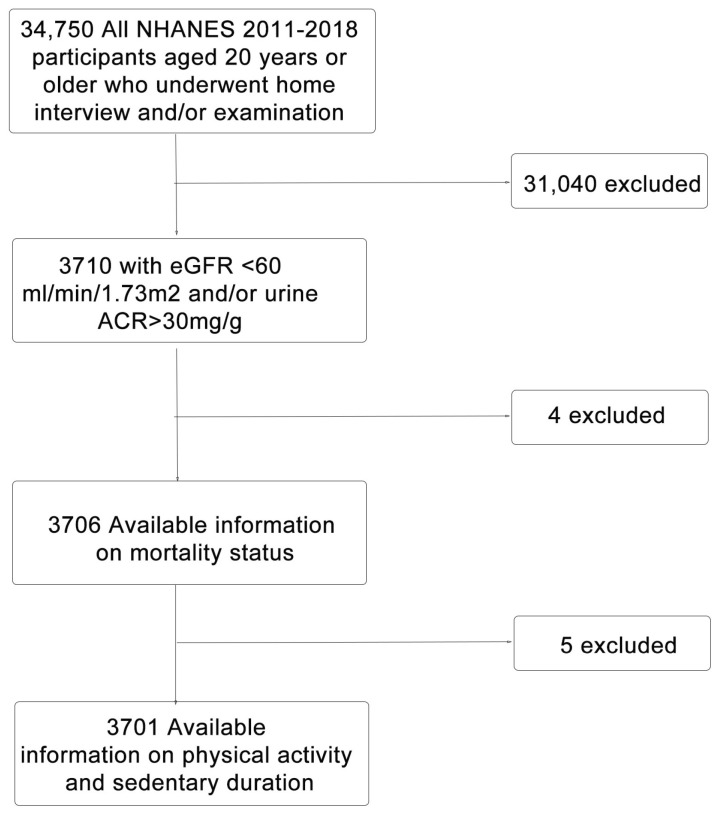
Flowchart of cumulative study participant exclusions.

Data were acquired from the NHANES survey, which were released by the Centers for Disease Control and Prevention (CDC). Details on data collection were introduced on the NHANES homepage (http://www.cdc.gov/nchs/nhanes.htm, accessed on 1 June 2022). Our study combined data from 4 cycles of NHANES spanning 2011–2018. The inclusion criteria for the current study were participants aged 20 years or older and having available measurements of renal function, including serum creatinine and urine albumin to creatinine ratio (ACR). We then excluded participants with missing information on mortality status or physical activity. Figure 1 illustrates the selection process of study groups. Every participant provided written informed consent, and the process was approved by the National Center for Health Statistics Institutional Review Board of the CDC.

### 2.2. Assessment of Renal Function

Serum creatinine and urine ACR were measured. Serum creatinine was used to calculate the estimated glomerular filtration rate (eGFR) with the CKD-EPI equation [10]. CKD was defined as an eGFR <60 mL/min/1.73 m^2^ and/or a urinary ACR >30 mg/g [11].

### 2.3. Assessment of Physical Activity

The “Physical activity” questionnaire was the data source. It was based on the Global Physical Activity Questionnaire and administered by trained interviewers [12]. Additional information about the questionnaire can be obtained at the World Health Organization website. The frequency and the time that respondents spend doing different types of physical activity, including sedentary activity, in a typical week/day were collected. The metabolic equivalent of task (MET) scores were assigned for each activity based on the NHANES guidelines [13]; that is, vigorous-intensity physical activity scores 8 MET; moderate-intensity physical activity, and walking or bicycling for transportation score 4 MET. Time spent in light activity and sedentary activities such as watching TV were not included in the MET calculation. Based on the United States physical activity guidelines (https://health.gov/sites/default/files/2019-09/paguide.pdf), we defined participants as extremely highly active (>1500 MET-min/week), highly active (>600, ≤1500 MET-min/week), low-active (>0, ≤600 MET-min/week) or inactive (0 MET-min/week).

### 2.4. Follow-Up Data

The study outcome was mortality during the follow-up period. Mortality status was collected from public-use linked mortality files updated with mortality follow-up data through 26 April 2022 (https://www.cdc.gov/nchs/data-linkage/mortality-public.htm).

### 2.5. Assessment of Demographic, Clinical, and Laboratory Measures

Demographic variables of the NHANES were collected, including age, gender, race/ethnicity, education, marital status, and family income-to-poverty ratio (PIR). Waist circumference, body mass index (BMI), and blood pressure were measured during the physical examination. Laboratory data of the NHANES were collected and used to assess the presence of comorbidities described as follows. Diabetes was defined as a glycated hemoglobin (HbAlc) ≥ 6.5%, a self-reported physician diagnosis of diabetes, or the current use of medications for diabetes or high blood sugar. Hypertension was defined as an average systolic blood pressure ≥ 140 mmHg and/or an average diastolic blood pressure ≥ 90 mmHg; the current use of antihypertensive medications or a self-reported physician diagnosis of hypertension. Participants were considered to have dyslipidemia if their total cholesterol was ≥ 240 mg/dL, or triglyceridemia ≥ 150 mg/dL, or low-density lipoprotein cholesterol (LDL-C) ≥ 130 mg/dL, or high-density lipoprotein cholesterol (HDL-C) < 40 mg/dL for men and < 50 mg/dL for women, or the reported use of cholesterol-lowering agents [14]. A history of coronary heart disease, angina, stroke, heart attack (myocardial infraction), congestive heart failure, and cancer were self-reported in the Medical Conditions Questionnaire. Smoking status was collected by self-report using a questionnaire: (1) have you smoked at least 100 cigarettes in your entire life? and (2) do you now smoke cigarettes? We divided smoking status into never a smoker, an ex-smoker, and a current smoker.

### 2.6. Statistical Analysis

All the analyses were performed with the statistical software packages R (R version 3.6.3 http://www.r-project.org, The R Foundation). In addition, sampling weights were used to produce nationally representative prevalence estimates for the non-institutionalized US population according to the NHANES introduction. Continuous variables were shown as a survey-weighted mean (SE). Categorical variables were shown as a survey-weighted percentage and missing values were categorized as a group. *p* values < 0.05 were considered statistically significant.

Schoenfeld residual plots were drawn to examine the proportional hazards assumption, and no violations were noted. Cox proportional hazards models were used to analyse the association between activities and study outcomes. Covariates were included as potential confounders in the final models if they changed the estimates of activities on mortality by more than 10% or were significantly associated with mortality status. For the analysis of the relationship between MET and mortality, the inactive group (0 MET-min/week) was used as the reference. Models were adjusted for age, race, education, marital status, PIR, smoking status, BMI, sedentary activity, and co-morbidities including diabetes, hypertension, dyslipidaemia, congestive heart failure, coronary artery disease, angina pectoris, myocardial infraction, stroke, and cancer. Hazards ratios (HRs) and 95% confidence intervals (CIs) were reported. To evaluate the dose–response relationship of MET (as continuous variables) with mortality risk, restricted cubic spline models were conducted. Additionally, in secondary analysis, we used a narrowed definition of CKD with an eGFR < 60 mL/min/1.73 m^2^. Similar analyses were performed as listed above and displayed in supplementary materials. Moreover, we performed additional analyses to examine the association between physical activity and mortality by CKD stage.

## 3. Results

### 3.1. Participant Characteristics

A total of 3701 individuals were evaluated, of which 43.2% were men (Table 1). The median age at study entry was 60.8 (0.4) years. Compared to those with a lower MET score, highly active CKD participants tended to be younger, male, more highly educated, and financially secure with a higher PIR. They were less likely to be diabetic, hypertensive, and had a lower prevalence of dyslipidaemia, cancer, stroke, advanced CKD stages, and cardiovascular disease including coronary artery disease, angina, and myocardial infraction. The patients with higher physical activity also tended to have higher hemoglobin levels, higher serum albumin levels, and lower HbA1c levels. The four groups did not differ significantly in smoking, blood pressure, serum cholesterol, serum triglyceride, serum LDL-C, and HDL-C.

### 3.2. Association between Physical Activity and Mortality According to Gender

**Figure 2:**
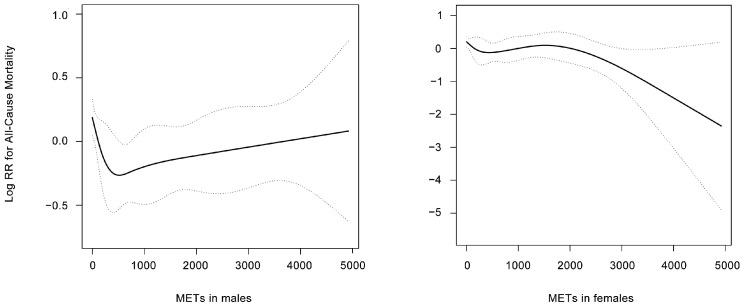
Restricted cubic spline model demonstrates the relationship between physical activity and all-cause mortality in males and females. The resulting figures show the predicted log RR (relative risk) in the y-axis and the MET (min/week) in the x-axis.

During the median follow up of 53.7 months, 694 all-cause deaths, 226 cardiovascular deaths, and 468 non-cardiovascular deaths were recorded. Multivariable Cox regression analyses were conducted to determine the risks of mortality across different physical activity categories (Table 2). After adjusting for confounding factors in male participants, compared to the physically inactive group (0 MET-min/week), the low-active group (>0, ≤600 MET-min/week) had lower risks for all-cause mortality (HR, 0.67; 95% CI, 0.48–0.93) and non-cardiovascular mortality (HR, 0.62; 95% CI, 0.41–0.92). Similar results were found in the highly active group (>600, ≤1500 MET-min/week); all-cause mortality reduced by 40% and non-cardiovascular mortality by 43%. However, the risks of all-cause mortality did not further decrease once physical activity surpassed 1500 MET-min/week, indicating a U-shaped association. Additionally, the association between physical activity and cardiovascular mortality was not significant in males. As for female participants, only the extremely highly active group was significantly associated with lower risks of all-cause mortality (HR, 0.59; 95% CI, 0.39–0.89) and cardiovascular mortality (HR, 0.40; 95% CI, 0.17–0.96). Restricted cubic spline analyses also confirmed a U-shaped association between MET (as a continuous variable) and mortality in males (Figure 2). Similar results were found when we narrowed the definition of CKD (Table S1). Moreover, the association between physical activity and mortality differed by the CKD stage (Table S2).

## 4. Discussion

In present study, we found different associations between physical activity and mortality in the male and female CKD population. A U-shaped association between higher physical activity and risks of all-cause mortality was confirmed among the males, while the beneficial effect on mortality was only observed in the females reporting extremely high physical activity (>1500 MET-min/week).

Apparently, the numbers of participants in the low-active group and highly active group were much smaller than the other two groups; however, the estimates in males and females were clearly different, and the results were not likely to be affected by relatively small event numbers. Herein, compared to inactive group, any levels of physical activity did reduce risks of all-cause mortality among the male patients. The association between physical activity and mortality among the CKD population is different from the general population. The 2008 Physical Activity Guidelines for Americans recommend adults should do at least 150 min a week of moderate-intensity or 75 min a week of vigorous-intensity activity; that is, 600 MET-min/week. Adults gain more extensive health and fitness benefits with even more physical activity. However, in the male CKD population, the benefits of physical activity on risk reduction of mortality appeared to reach a threshold of 1500 MET-min/week and did not further increase in the extremely high physical activity group. Our study suggested greater physical activity was associated with lower all-cause mortality in CKD patients in a dose–response manner. In accordance to our findings, a report on the patients with renal insufficiency (eGFR<60 mL/min/1.73 m^2^) from the NHANES III cohort demonstrated a remarkable decline in the risk of mortality [15]. However, we did not find a beneficial effect of physical activity on cardiovascular mortality in males, which was not anticipated. Previous study suggested that males tend to overestimate their physical activity levels [16]; thus, the results may be biased by the nature of self-reported data.

As for females, we confirmed a survival benefit of extremely high physical activity. The high intensity may be the key as earlier reports found a moderate intensity of physical activity failed to improve lipid profiles in women and CKD patients, while high intensity exercise induces substantial benefits on aerobic fitness [17,18,19]. Conversely, Molsted et al. demonstrated an association between moderate physical activity and reduced all-cause mortality in women [20]. We noticed that patients in this study experienced a more advanced stage of CKD with a median eGFR of 22 mL/min/1.73 m^2^, while the majority of our participants were at stage 1–3. Besides, Molsted et al. reported a relatively low number of women with high levels of physical activity (*n* = 9). Diverse effects of physical activity were found in women with or without obese, diabetes, or hypertension [18,21]. Some indicated physical activity strongly affected parameters related to cardiovascular function, with large differences in the magnitude by gender, while others reported opposite, even null correlation [17,22,23]. This heterogeneity can be attributable to age-related hormones, cardiovascular adaptability, and baseline health conditions. In the present study, the majority of the female participants were postmenopausal. Previous reports suggested that postmenopausal women had a blunted response to exercise-induced cardiovascular adaptations, partly due to the fact that estrogen status is pivotal to exercise-induced improvements in endothelial function and other cardiovascular risk factors in women [24,25]. In addition, physical activity interacts with steroid hormones in different ways between males and females. Specifically, males who engage in a higher intensity of physical activity have higher levels of testosterone, while females report a U-shaped association between hormones and physical activity [26,27]. Moreover, fundamental differences in cardiovascular regulation could further evoke the gender specificity of long-term benefits of physical activity. For example, autonomic function is impaired in middle-aged women, but not men [28]. Muscle sympathetic nerve activity is found to increase to a greater extent in women compared to men, leading to adverse effects on blood pressure and the cardiovascular system [29]. Reduced left ventricular relaxation is more significant in women than in men [30]. Unfortunately, it is hard to depict a clear picture as there is limited information about the possible mechanisms related to gender disparities in exercise.

Chronic exertional fatigue and exercise intolerance feature a majority of the CKD patients. It becomes apparent in the early stages and worsens as disease severity increases [31]. The underlying mechanisms are multifaceted, involving pulmonary dysfunction, cardiac limitation, impaired nerve activity, anemia, and iron deficiencies [32]. Physical inactivity is highly prevalent across the spectrum of CKD. We found a much higher proportion of physically inactive patients, compared to a previous study on the US CKD patients collected between 1988 and 1994 (40% vs. 28%) [15]. Since 2012, KDIGO guidelines recommend that patients should engage in moderate physical activity [5]. Apparently, this needs to be reinforced. Data from previous studies have provided inconsistent evidence, indicating a susceptible role of physical activity. In contrast, physical inactivity plays a more substantial role on clinical outcomes. Although it is hard to provide concrete guidance about the types and amounts of physical activity appropriate for patients’ abilities, according to current studies, some physical activity is better than none. CKD patients participating in any amount of physical activity will gain some health benefits.

The present study has several limitations. Firstly, residual confounding cannot be ruled out. Secondly, part of our variables are based on self-reported data, which could have led to recall bias. Furthermore, we did not have longitudinal data on patients’ physical activity during the follow-up period. Despite these limitations, the standardized and rigorous procedures for data collection in NHANES guaranteed the reliability.

## 5. Conclusions

In conclusion, the association between physical activity and mortality differs between male and female CKD patients. For male patients, compared to physical inactivity, any amount of physical activity is associated with lower risks of all-cause mortality. Additional survival benefits may not be achieved in males when the physical activity volume exceeds 1500 MET-min/week. For female patients, only the extremely highly active group shows the significant association. A minimum volume of 1500 MET-min/week is recommended to postpone mortality.

## Figures and Tables

**Table 1:** Baseline characteristics of study participants by physical activity.

Characteristics	Total *n* = 3701	Physical Activity MET-Min/Week				*p* Value
		Inactive 0 (*n* = 1466)	Low-Active >0, ≤600 (*n* = 612)	Highly Active >600, ≤1500 (*n* = 501)	Extremely Highly Active >1500 (*n* = 1122)	
Demographics						
Age, years	60.8 (0.4)	66.1 (0.6)	61.8 (0.7)	60.6 (1.1)	54.6 (0.8)	<0.001
Gender						<0.001
Male	43.2	38.1	36.5	35.5	55.5	
Female	56.8	61.9	63.5	64.5	44.5	
Race						<0.001
Mexican American	7.3	8.0	6.3	4.3	8.4	
Other Hispanic	5.0	5.6	3.6	3.7	5.5	
Non-Hispanic White	67.6	67.8	64.6	72.3	66.8	
Non-Hispanic Black	12.2	11.8	14.1	11.5	12.1	
Other race	7.9	6.7	11.2	8.2	7.2	
PIR	2.7 (0.1)	2.4 (0.1)	2.8 (0.1)	3.1 (0.1)	2.7 (0.1)	<0.001
Education						<0.001
<High school	19.8	26.8	16.5	13.6	16.6	
High school	25.2	26.0	24.6	25.7	24.6	
>High school	54.8	46.9	59.0	60.7	58.6	
Marital status						<0.001
Married/living with partner	57.0	53.1	57.2	60.0	59.7	
Divorced/separated/widowed	31.6	38.2	32.1	30.6	24.6	
Never married	11.3	8.5	10.7	9.3	15.7	
Smoking						0.071
Never smoker	50.6	47.2	54.1	52.0	51.6	
Ex-smoker	33.7	37.0	32.2	35.1	30.5	
Current smoker	15.7	15.8	13.7	12.9	17.9	
BMI, kg/m^2^	30.5 (0.2)	31.2 (0.3)	30.7 (0.4)	29.8 (0.5)	30.0 (0.3)	0.018
Sedentary activity, min/day	405.4 (5.6)	471.3 (9.3)	414.7 (10.4)	405.9 (12.5)	332.2 (7.4)	<0.001
CKD awareness	11.0	13.5	11.7	11.1	8.0	0.036
CKD						<0.001
Stage 1 or 2	51.8	43.2	48.9	48.9	63.4	
Stage 3	44.1	49.9	47.6	48.0	34.6	
Stage 4	2.9	4.9	1.9	2.0	1.5	
Stage 5	1.3	2.0	1.6	1.1	0.5	
Comorbidity						
Diabetes	31.6	39.5	31.3	31.3	23.4	<0.001
Hypertension	60.6	70.1	64.2	60.3	48.8	<0.001
Dyslipidaemia	67.4	70.1	70.0	64.9	64.3	0.057
Congestive heart failure	9.4	14.7	7.9	9.3	4.7	<0.001
Cardiovascular disease						
Coronary artery disease	10.3	13.1	10.2	10.6	7.2	0.002
Angina pectoris	6.0	7.4	6.5	6.4	3.9	0.008
Myocardial infraction	9.3	12.0	7.4	10.1	6.8	0.003
Stroke	8.5	14.3	6.8	5.6	4.3	0.001
Cancer	19.6	23.0	20.8	21.0	14.5	0.002
Biochemical data						
Systolic blood pressure, mmHg	119.4 (0.5)	120.8 (0.8)	118.6 (1.1)	118.1 (1.6)	119.0 (0.9)	0.287
Diastolic blood pressure, mmHg	66.2 (0.4)	66.6 (0.5)	66.8 (0.9)	65.8 (1.0)	65.8 (0.5)	0.601
Hemoglobin, g/dL	13.7 (0.0)	13.4 (0.1)	13.7 (0.1)	13.6 (0.1)	14.1 (0.1)	<0.001
Albumin, g/L	41.0 (0.2)	40.7 (0.2)	41.4 (0.2)	41.5 (0.2)	42.2 (0.2)	<0.001
Total cholesterol, mg/dL	191.7 (1.2)	188.1 (1.5)	190.9 (3.1)	194.1 (2.9)	194.5 (2.3)	0.124
Triglyceride, mg/dL	136.1 (3.6)	184.1(5.9)	175.5 (7.3)	160.5 (8.3)	166.1 (5.3)	0.086
LDL-C, mg/dL	107.7 (1.1)	105.5 (1.8)	106.6 (3.2)	109.7 (3.0)	109.6 (2.7)	0.514
HDL-C, mg/dL	53.1 (0.5)	51.5 (0.9)	52.9 (1.0)	55.2 (1.1)	53.9 (0.8)	0.074
HbA1c	6.2 (0.1)	6.4 (0.1)	6.1 (0.1)	6.2 (0.1)	6.1 (0.1)	<0.001

MET, metabolic equivalent of task; PIR, family poverty income ratio; BMI, body mass index; CKD, chronic kidney disease; LDL-C, low-density lipoprotein cholesterol; HDL-C, high-density lipoprotein cholesterol; eGFR, estimated glomerular filtration rate; ACR, albumin creatinine ratio; HbA1c, glycohemoglobin. Values for categorical and continuous variables are expressed as % and mean (SE), respectively.

**Table 2:** Estimated hazard ratios from the Cox regression analyses of the association between mortality and the physical activity level in male and female.

	Physical Activity, Met (Mins/Week)			
	0 (*n* = 1461)	>0, ≤600 (*n* = 607)	>600, ≤1500 (*n* = 496)	>1500 (*n* = 1117)
Male				
All-cause mortality				
Event, *n*	206	57	38	84
HR (95% CI)	Ref	0.67 (0.48, 0.93)	0.60 (0.41, 0.88)	0.65 (0.48, 0.88)
Cardiovascular mortality				
Event, *n*	65	18	12	32
HR (95% CI)	Ref	0.81 (0.46, 1.44)	0.68 (0.34, 1.37)	0.93 (0.55, 1.55)
Non-cardiovascular mortality				
Event, *n*	141	39	26	52
HR (95% CI)	Ref	0.62 (0.41, 0.92)	0.57 (0.36, 0.90)	0.56 (0.39, 0.81)
Female				
All-cause mortality				
Event, *n*	205	41	29	34
HR (95% CI)	Ref	0.73 (0.50, 1.05)	0.81 (0.54, 1.23)	0.59 (0.39, 0.89)
Cardiovascular mortality				
Event, *n*	76	8	8	7
HR (95% CI)	Ref	0.50 (0.23, 1.06)	0.63 (0.29, 1.37)	0.40 (0.17, 0.96)
Non-cardiovascular mortality				
Event, *n*	129	33	21	27
HR (95% CI)	Ref	0.85 (0.55, 1.30)	0.87 (0.53, 1.43)	0.67 (0.42, 1.07)

Adjusted for age, race, education, marital status, PIR, smoking and BMI, sedentary activity, and comorbidities including diabetes, hypertension, dyslipidaemia, congestive heart failure, cardiovascular disease, and cancer.

## Data Availability

The datasets generated and/or analysed during the current study are available on the NHANES (https://www.cdc.gov/nchs/nhanes/index.htm, accessed on 1 June 2022).

## Supplementary Materials

The following supporting information can be downloaded at: https://www.mdpi.com/article/10.3390/jcm12030779/s1, Table S1: Estimated hazard ratios from the Cox regression analyses of the association between. Table S2: Estimated hazard ratios from the Cox regression analyses of the association between mortality and the physical activity level in male and female with different CKD stages.
