# Supplementary material for: Gender Differences in the Association between Physical Activity and Mortality in Chronic Kidney Disease: Results from the National Health and Nutrition Examination Survey (2011–2018)
Source: J Clin Med. 2023 Jan 18;12(3):779. doi: 10.3390/jcm12030779 (PMC9918191; doi:10.3390/jcm12030779)
Supplement: Supplementary file 1 [file jcm-12-00779-s001.zip › jcm-2101298-supplementary.pdf]

Table S1. Estimated hazard ratios from the Cox regression analyses of the association between mortality and the physical activity level in male and female with an eGFR<60ml/min/1.73m^2.

	Physical activity, MET(mins/week)			
	0 (n=834)	>0, ≤600 (n=307)	>600, ≤1500 (n=237)	>1500 (n=412)
Male				
All-cause mortality				
Event, n	153	35	21	49
HR (95% CI)	Ref	0.56 (0.37, 0.86)	0.45 (0.27, 0.75)	0.61 (0.42, 0.90)
Cardiovascular mortality				
Event, n	51	15	9	18
HR (95% CI)	Ref	0.93 (0.49, 1.79)	0.74 (0.33, 1.68)	0.79 (0.41, 1.52)
Non-Cardiovascular mortality				
Event, n	102	20	12	31
HR (95% CI)	Ref	0.42 (0.23, 0.75)	0.35 (0.18, 0.69)	0.56 (0.35, 0.88)
Female				
All-cause mortality				
Event, n	150	28	24	16
HR (95% CI)	Ref	0.61 (0.39, 0.98)	1.07 (0.67, 1.71)	0.42 (0.23, 0.76)
Cardiovascular mortality				
Event, n	54	7	6	2
HR (95% CI)	Ref	0.48 (0.21, 1.12)	0.67 (0.27, 1.69)	0.10 (0.01, 0.74)
Non-Cardiovascular mortality				
Event, n	96	21	18	14
HR (95% CI)	Ref	0.67 (0.38, 1.17)	1.22 (0.70, 2.12)	0.56 (0.30, 1.08)

Table S2. Estimated hazard ratios from the Cox regression analyses of the association between mortality and the physical activity level in male and female with different CKD stages.

	CKD stage 1 or 2		CKD stage 3	
All-cause mortality	Male	Female	Male	Female
MET				
0	Ref	Ref	Ref	Ref
>0, ≤600	0.83 (0.48, 1.43)	1.08 (0.56, 2.08)	0.66 (0.43, 1.02)	0.65 (0.40, 1.07)
>600, ≤1500	0.88 (0.48, 1.63)	0.46 (0.18, 1.18)	0.38 (0.23, 0.65)	1.00 (0.60, 1.65)
>1500	0.79 (0.48, 1.29)	0.95 (0.51, 1.75)	0.63 (0.43, 0.91)	0.41 (0.22, 0.76)
Cardiovascular mortality				
MET				
0	Ref	Ref	Ref	Ref
>0, ≤600	0.44 (0.10, 1.90)	0.42 (0.06, 3.11)	1.16 (0.60, 2.23)	0.44 (0.16, 1.17)
>600, ≤1500	0.91 (0.21, 3.89)	0.37 (0.09, 1.59)	0.76 (0.35, 1.67)	0.80 (0.29, 2.22)
>1500	2.02 (0.74, 5.52)	1.15 (0.44, 3.03)	0.82 (0.44, 1.52)	0.22 (0.05, 0.95)
Non-cardiovascular mortality				
MET				
0	Ref	Ref	Ref	Ref
>0, ≤600	0.96 (0.53, 1.76)	1.28 (0.62, 2.63)	0.46 (0.25, 0.84)	0.76 (0.43, 1.35)
>600, ≤1500	0.95 (0.48, 1.90)	0.42 (0.12, 1.43)	0.24 (0.11, 0.52)	1.09 (0.60, 1.96)
>1500	0.57 (0.31, 1.04)	091 (0.43, 1.93)	0.54 (0.34, 0.87)	0.48 (0.24, 0.96)
